# Prevalence of peri‐implantitis in a sample of HIV‐positive patients

**DOI:** 10.1002/cre2.469

**Published:** 2021-07-20

**Authors:** Luca Casula, Andrea Poli, Tommaso Clemente, Giulia Artuso, Paolo Capparé, Enrico F. Gherlone

**Affiliations:** ^1^ Oral Surgery Resident, Department of Dentistry Vita‐Salute San Raffaele University Milan Italy; ^2^ BioEngineering IRCCS San Raffaele Scientific Institute Milan Italy; ^3^ Infectious Diseases IRCCS San Raffaele Scientific Institute Milan Italy; ^4^ Private practice Milan Italy; ^5^ Dental School Vita‐Salute San Raffaele University Milan Italy; ^6^ Department of Dentistry Vita‐Salute San Raffaele University and IRCCS San Raffaele Hospital Milan Italy

**Keywords:** all‐on‐4, HIV‐positive patients, mucositis, peri‐implantitis

## Abstract

**Objectives:**

This study aimed to assess the prevalence of peri‐implantitis in human immunodeficiency virus (HIV)‐positive patients and the presence of a possible correlation between the immunological profile and serological values, of peri‐implantitis, and of possible differences between all‐on‐4 and single crown/bridge prostheses.

**Subjects and methods:**

This retrospective study included 58 adult HIV‐positive patients (222 implants) with either all‐in‐4 prostheses or single crowns/bridges on at least one dental implant loaded for more than a year who were followed for 3 year (mean follow‐up). Data pertaining to the probing pocket depth (PPD), bleeding on probing, and immunological and systemic profile were collected.

**Results:**

Patients with single crown/bridge implant rehabilitation showed higher prevalence of peri‐implantitis (34%) than patients with all‐on‐4 rehabilitation (0%) (*p* = 0.012). Patients with all‐on‐4 rehabilitation were significantly older than those with single crowns/bridges (*p* = 0.004). Patients with peri‐implantitis had implants for a significantly longer duration than those without (*p* = 0.001), implying that the probability of peri‐implantitis increases as the age of implant increases.

**Conclusions:**

The prevalence of peri‐implantitis was 26% in the HIV‐positive patients population. No correlation was found between patients' immunological and serological factors and peri‐implantitis. The most important risk factor for peri‐implantitis and mucositis was implant age.

## 1 INTRODUCTION

Dental implants are being increasingly used for the replacement of missing teeth and have a high survival rate (> 10 years) (Renvert et al., 2018). Despite their long‐term success rate, dental implants are subject to biological complications characterized by inflammation of the soft tissues and bone in contact with the implant and its prosthetic components, caused primarily by the accumulation of bacterial plaque (Costa et al., 2012; Vignoletti et al., 2019), and bearing a pathogenic mechanism similar to periodontitis (Heitz‐Mayfield & Lang, 2010). These biological complications are classified as peri‐implant mucositis and peri‐implantitis (Lang et al., 2011; Mombelli et al., 2012; Araujo et al., 2018, Renvert et al., 2014).

Peri‐implant mucositis is inflammation of the mucosa around the implant and is not associated with the loss of supporting bone. In contrast, peri‐implantitis is characterized by inflammation of the peri‐implant mucosa associated with a progressive loss of supporting bone (Heitz‐Mayfield & Salvi, 2018; Lindhe et al., 2008; Schwarz et al., 2018; Zitzmann & Berglundh, 2008).

In addition to bacterial plaque, potential risk factors and indicators have been associated with peri‐implantitis, such as history of periodontitis, current or past smoking habits, absence of an adequate amount of keratinised mucosa, diabetes mellitus, iatrogenic factors, excess cement in the peri‐implant gingival sulcus, factors related to the type and number of implants, genetic factors, and/or systemic conditions (Ferreira et al., 2006; Gruica et al., 2004; Hamdy & Ebrahem, 2011; Karoussis et al., 2003; Lachmann et al., 2007; Laine et al., 2006; Lindhe et al., 2008; Roos‐Jansaker, Lindahl, et al., 2006; Roos‐Jansaker, Renvert, et al., 2006; Souza et al., 2016; Ueno et al., 2016).

Systemic conditions, other than diabetes, correlated with peri‐implantitis include cardiovascular diseases, rheumatoid arthritis, osteoporosis, osteopenia, thyroid disease, hepatitis, radiation, and/or chemotherapy (Dalago et al., 2017; de Araujo Nobre et al., 2015; Dvorak et al., 2011; Marrone et al., 2013). However, these diseases have not been proved to be correlated to peri‐implantitis, and no cause‐effect relationship has been found, as has been for diabetes mellitus (de Araújo Nobre & Maló, 2017).

The presence of inflammation in peri‐implant tissues is a common factor between peri‐implantitis and mucositis. Hence, determination of bone loss through radiographs is essential for distinguishing between the two (Renvert et al., 2018; Schwarz et al., 2018).

However, functional bone loss between 0.5 and 2 mm within 1 year from the loading of implants is considered physiological, and occurs as a result of the natural healing process (Lindquist et al., 1996; Mombelli et al., 1987). Any other radiographic evidence of bone loss should be considered pathological (Renvert et al., 2018).

The progressive loss of bone around dental implants can be quantified by comparing a current radiograph with one obtained at least 1 year after implant loading. In absence of initial radiographs, diagnosis of peri‐implantitis is based on: bone levels > 3 mm apical to the most coronal portion of the intra‐osseous part of the implant and/or probing depths of >6 mm (Fransson et al., 2005; Koldsland et al., 2010; Papantonopoulos et al., 2015; Renvert et al., 2018).

Numerous cross‐sectional studies have reported the prevalence of peri‐implantitis; a meta‐analysis of 11 studies published in 2015 reported the average prevalence of peri‐implantitis as 22% (range, 1%–47%) (Derks & Tomasi, 2015).

Dental implants have been widely used even in human immunodeficiency virus (HIV)‐positive patients with results similar to healthy subjects, owing to the introduction of the highly active antiretroviral therapy, which has extended the average life expectancy of such patients (Capparé et al., 2019; Dios et al., 1999; Gherlone et al., 2016a; Gherlone et al., 2016b; Oliveira et al., 2011; Rubinstein et al., 2019; Sabbah et al., 2019).

A recent study that analyzed patients with systemic diseases including HIV infection reported the prevalence of peri‐implantitis as 15.3% (921 patients out of 5988). However, HIV infection was not analyzed as a single factor (de Araújo Nobre & Maló, 2017).

The analysis of the immunological profile and serological values in a population of HIV‐positive patients who had undergone prosthetic rehabilitation with implants (7 years of observation) revealed a statistically significant correlation (*p* = 0.009) between the viral load of patients and early implant failure due to infection and lack of osseointegration (Capparé et al., 2019). Additionally, a statistically significant correlation was found between the CD4+/CD8+ ratio and late implant failure, possibly due to the presence of peri‐implantitis (defined as a progressive loss of bone with signs of infection around the implant) (Capparé et al., 2019). Other authors did not find a correlation between implant survival (1 year follow‐up) and levels of CD4+ cells (CD4 < 749.5 or CD4 > 749.5) ( Gherlone et al., 2016a; Gherlone et al., 2016b). However, to date, data for understanding the interaction between immune status and success of implant therapy are not sufficient (Duttenhoefer et al., 2019).

The aim of the present study was to assess the prevalence of peri‐implant disease in an HIV‐positive population and to evaluate correlation between the peri‐implant disease and the population immune status.

## 2 MATERIALS AND METHODS

### 2.1 Type and sample of the study

This retrospective monocentric observational study assessed the prevalence of peri‐implant disease in HIV‐positive patients treated at the department of dentistry of our hospital. At the same hospital, the HIV‐positive patients were undergoing antiretroviral treatment. This study included 58 adult patients with at least one dental implant and a total of 222 implants followed for at least 1 year. Demographic, clinical, and laboratory information of the patients was collected.

All procedures were approved by the Ethics Committee of University Vita‐Salute, San Raffaele Hospital, Milan (on 15/7/2020 with number of protocol “PERIHIV2”—EC Reg. N. 133/INT/2020). Appropriate informed consent forms were provided to all patients and were signed if the patients agreed to participate in the study.

This study has been reported according to the STrengthening the Reporting of OBservational studies in Epidemiology (STROBE) guidelines and checklist.

### 2.2 Study design

All included patients were divided into two groups, according to the type of prosthesis inserted after implant placement (all‐on‐4 prosthesis vs. single crowns\bridges), to assess the differences between the two types of prostheses in the inflammatory state, and therefore, in the presence of mucositis and peri‐implantitis as well as in the variables analyzed.

As shown in Table 1, the data related to the immunological and systemic profile of the patients (HIVRNA load, CD4+ level, CD8+ level, hemoglobin level, and platelet count) were collected through the database of the hospital.

**TABLE 1:** Collected data

Patient's age (years)	CD4+/CD8+ ratio at the last visit
Patient's sex	Baseline hemoglobin level (mg/dL)
Years of HIV infection	Hemoglobin level at the last visit (mg/dL)
Years of antiretroviral therapy	Baseline platelet count (×10^9^/L)
Baseline HIVRNA >50 cp/mL	Platelet count at the last visit (×10^9^/L)
HIVRNA >50 cp/mL at the last visit	Number of implants
Baseline CD+ levels (cells/μL)	Implant diameter (mm)
CD4+ levels at the last visit (cells/μL)	Implant age (months)
Average CD4+ level during follow‐up (cells/μL)	Presence of peri‐implantitis
Baseline CD8+ levels (cells/μL)	Probing pocket depth (mm)
CD8+ level at the last visit (cells/μL)	Presence of mucositis
Baseline CD4+/CD8+ ratio	

*Note*: HIV: human immunodeficiency virus.

### 2.3 Case definition for peri‐implant condition

The clinical and radiological data for the diagnosis of peri‐implant disease were collected according to the criteria laid in the Periodontology World Workshop in 2017 by Renvert et al. (2018).

#### 2.3.1 Clinical examination data

- Peri‐implant mucositis: It was diagnosed on the basis of the following criteria:

1. Visual inspection demonstrating the following signs of inflammation in the peri‐implant region: red as opposed to pink color, swollen tissues as opposed to no swelling, and soft as opposed to firm tissue consistency.
2. Presence of profuse (line or drop) bleeding and/or suppuration on probing.
3. An increase in probing pocket depths (PPDs) compared to baseline.

- Peri‐implantitis: It was diagnosed on the basis of the following criteria:

1. Evidence of visual inflammatory changes in the peri‐implant soft tissues combined with bleeding on probing and/or suppuration;
2. Increasing PPDs as compared to measurements obtained at placement of the supra‐structure; and
3. Progressive bone loss in relation to the radiographic bone level assessment at 1 year following the delivery of the implant‐supported prosthetics reconstruction; and
4. In the absence of initial radiographs and probing depths, radio‐graphic evidence of bone level ≥ 3 mm and/or probing depths ≥6 mm in conjunction with profuse bleeding represents peri‐implantitis.

#### 2.3.2 Radiological examination data

Intraoral periapical radiographs are considered gold standard for radiological evaluation and were used in this study. The radiographic data were collected from the records of the patient. The position of the marginal bone was measured manually on an ultraspeed radiographic film (Ultra speed, Kodak, USA) using a dental caliper (0–10 mm). The distance to the marginal bone was measured from the mesial and distal aspects of the implants. The implant platform was used as a reference for the measurements. Two vertical lines were drawn parallel to a vertical line passing through the centre of the implant. The largest value was considered for statistical analysis. All assessments were performed by a single investigator (LC).

Peri‐implantitis was assessed by radiographic examination data as progressive bone loss in relation to the radiographic bone level assessment at 1 year following the delivery of the implant‐supported prosthetics reconstruction and in the absence of initial radiographs, radiographic evidence of bone level ≥3 mm.

#### 2.3.3 Inclusion criteria

All patients (>18 years old), who were undergoing antiretroviral therapy at the same hospital, who had undergone prosthetic rehabilitation using one or more dental implants, with at least 1 year of follow‐up after loading, and who had returned for a maintenance appointment were considered for inclusion.

#### 2.3.4 Exclusion criteria

Patients who discontinued antiretroviral therapy, patients not undergoing therapy, patients whose previous radiographs and data on bleeding on probing and PPD at gingival level were unknown, patients with decompensated systemic diseases (for example, decompensated diabetes mellitus), patients treated with drugs that affect the bone turnover (e.g., bisphosphonates), and patients under 18 years of age were not included in this study.

### 2.4 Statistical analysis

A descriptive analysis was performed to assess all the obtained data. Continuous quantitative variables were described as medians and interquartile ranges, and categorical qualitative variables as frequencies and percentages (%). The continuous variables are graphically represented using boxplots [the box shows the first (Q1), second (median), and third quartiles (Q3), and as whiskers, the values correspond to 1.5 times <Q1 and 1.5 times> Q3] and categorical variables using bar graphs. The quantitative variables were compared using the non‐parametric Mann–Whitney test and qualitative variables using the chi‐square test or Fisher's exact test. Stepwise multivariate logistic regression analysis was used to calculate the adjusted risks (odds), respective 95% confidence intervals, and probability (*p*‐value) of developing peri‐implantitis and mucositis. All analyses were performed using SAS for Windows Software (Version 9.4, SAS Institute). All statistical tests were applied to 2 sails, and values were considered significant if the calculated probability was <0.05. The following two types of analyses were performed: the first analyzed the variables individually in the entire study population and the other in the population divided into two groups according to the type of implant‐prosthetic rehabilitation performed.

## 3 RESULTS

The prevalence of peri‐implantitis in the HIV‐positive patient population was 26%. Table 2 shows the comparisons of individual variables (characteristics) between the patients of the two groups. For the first variable “age,” the results showed that patients with all‐on‐4 prosthetic rehabilitation were significantly older than those with single crowns/bridges (*p* = 0.004). For the second variable “years of HIV infection,” the results indicated that patients with all‐on‐4 prosthetic rehabilitation were infected with HIV for significantly greater number of years than those with single crowns/bridges (*p* = 0.003). For the variable “number of implants,” patients with all‐on‐4 prosthetic rehabilitation had significantly greater number of implants than those with single crowns/bridges (*p* = 0.0001). This finding was expected and is obvious considering that the all‐on‐4 protocol requires the insertion of four implants. A statistically significant difference was observed in the prevalence of “peri‐implantitis” between the two groups (*p* = 0.012). In fact, no patient with all‐on‐four prosthetic rehabilitation exhibited any sign of peri‐implantitis, while in patients with single crowns/bridges, the prevalence was 34%. In terms of “PPD,” which is directly correlated with peri‐implantitis, it was significantly lower in patients with all‐on‐4 prosthetic rehabilitation than that in patients with single crowns/bridges (*p* = 0.0001).

**TABLE 2:** Patient characteristics based on the type of implant‐prosthetic rehabilitation

Characteristics	Overall	All‐on‐4	Single crown/bridge	*p*‐value
	(*n* = 58)	(*n* = 14)	(*n* = 44)	
Age (years)	55 (50–58)	58 (56–61)	53 (49–57)	0.004
Male sex	44 (76%)	11 (79%)	33 (75%)	0.999
Years of HIV infection	19.1 (9.7–27.1)	27.2 (21.1–31.1)	17.5 (8.0–21.5)	0.003
Years of antiretroviral therapy	17.9 (9.3–21.7)	21.7 (14.8–22.9)	14.9 (7.9–20.2)	0.014
Baseline HIVRNA >50 cp/mL	1 (2%)	0	1 (2%)	0.999
HIVRNA >50 cp/mL at the last visit	1 (2%)	0	1 (2%)	0.999
Baseline CD4+ level (cells/μL)	682 (588–1024)	907 (653–1030)	657 (588–1000)	0.418
CD4+ level at the last visit (cells/μL)	717 (509–1001)	884 (608–1247)	700 (506–963)	0.211
Average CD4+ level during follow‐up (cells/μL)	758 (548–1007)	920 (595–1287)	721 (539–957)	0.247
Baseline CD8+ level (cells/μL)	839 (613–1433)	961 (552–1433)	828 (621–1282)	0.981
CD8+ level at the last visit (cells/μL)	858 (539–1181)	958 (516–1264)	819 (545–1139)	0.629
Baseline CD4+/CD8+ ratio	0.78 (0.67–1.06)	0.77 (0.67–0.93)	0.81 (0.67–1.10)	0.922
CD4+/CD8+ ratio at the last visit	0.87 (0.64–1.17)	0.87 (0.65–1.11)	0.86 (0.63–1.24)	0.999
Baseline hemoglobin level (mg/dL)	15.0 (14.3–15.7)	14.7 (14.3–15.3)	15.1 (14.2–15.8)	0.307
Hemoglobin level at the last visit (mg/dL)	15.4 (13.6–15.9)	15.2 (13.7–15.7)	15.4 (13.2–15.9)	0.578
Baseline platelet count (**×**10^9^/L)	217 (174–258)	210 (174–258)	220 (174–264)	0.888
Platelet count at the last visit (**×**10^9^/L)	220 (184–261)	236 (176–269)	217 (184–261)	0.760
Number of implants	4 (1–5)	8 (5–8)	2 (1–4)	<0.0001
1	15 (26%)	0	15 (34%)	<0.0001
2	8 (14%)	0	8 (18%)	
3	6 (10%)	0	6 (14%)	
4	12 (21%)	3 (22%)	9 (20%)	
5	3 (5%)	1 (7%)	2 (5%)	
6	2 (3%)	0	2 (5%)	
7	1 (2%)	0	1 (2%)	
8	10 (17%)	9 (64%)	1 (2%)	
10	1 (2%)	1 (7%)	0	
Implant diameter (mm)	3.8 (3.8–3.8)	3.8 (3.8–3.8)	3.8 (3.8–4.5)	0.096
Implant age (months)	38 (23–48)	28.5 (22–48)	39 (24.5–47.5)	0.542
Peri‐implantitis	15 (26%)	0	15 (34%)	0.012
Probing pocket depth (mm)	3.0 (2.0–5.0)	1.5 (1.0–3.0)	4.0 (3.0–5.0)	0.0001
Mucositis	36 (62%)	10 (71%)	26 (59%)	0.533

*Note*: Data are presented as median and interquartile ranges or frequencies and percentages. HIV: human immunodeficiency virus.

The variables related to the patients' immunological and serological profiles (CD4+ and CD8+ levels and viremia) did not correlate with the type of prosthetic rehabilitation performed.

Table 2 shows the comparison of variables between patients with and without peri‐implantitis. Statistically significant differences were found between the two groups (patients with and without periodontitis) for the variables “duration of implant function” (*p* = 0.001), “implants for all‐on‐4 prosthetic rehabilitation” (*p* = 0.012), and “PPD” (*p* = 0.0001).

Patients with all‐on‐4 prosthetic rehabilitation were not affected by peri‐implantitis. Therefore, statistically significant differences were observed between patients with and without peri‐implantitis for the variable “implants for all‐on‐4 prosthetic rehabilitation” (*p* = 0.012).

For the variable ‘duration of implant function’, the results showed that patients with peri‐implantitis had implants for a significantly longer duration than those without (*p* = 0.001) or that the probability of peri‐implantitis increases as the age of the implant increases.

As evident from Table 3, PPDs in patients with peri‐implantitis were significantly higher than those in patients without peri‐implantitis (*p* = 0.0001). This is obvious as the diagnosis of peri‐implantitis is based on increase in PPDs. The variables related to the patients' immunological profile (CD4+ and CD8+ levels and viremia) did not correlate with peri‐implantitis.

**TABLE 3:** Patient characteristics based on the presence of peri‐implantitis

Characteristic	Peri‐implantitis	Healthy	*p*‐value
	(*n* = 15)	(*n* = 43)	
Age (years)	54 (50–58)	56 (50–59)	0.534
Male sex	11 (77%)	33 (73%)	0.999
Years of HIV infection	19.1 (8.0–20.4)	19.6 (11.8–28.1)	0.177
Years of antiretroviral therapy	16.8 (8.0–20.1)	18.2 (9.4–22.6)	0.370
Baseline HIVRNA >50 cp/mL	0	1 (2%)	0.999
HIVRNA >50 cp/mL at the last visit	1 (7%)	0	0.259
Baseline CD4+ level (cells/μL)	656 (684–777)	755 (588–1030)	0.418
CD4+ level at the last visit (cells/μL)	884 (608–1247)	725 (491–1043)	0.957
Mean CD4+ level during follow‐up (cells/μL)	721 (582–879)	774 (523–1044)	0.779
Baseline CD8+ level (cells/μL)	769 (613–1213)	845 (612–1501)	0.643
CD8+ level at the last visit (cells/μL)	826 (466–1138)	875 (624–1264)	0.345
Baseline CD4+/CD8+ ratio	0.90 (0.70–1.36)	0.77 (0.67–1.01)	0.367
CD4+/CD8+ ratio at the last visit	0.98 (0.67–1.68)	0.86 (0.63–1.09)	0.318
Baseline hemoglobin level (mg/dL)	15.1 (13.0–15.6)	15.0 (14.3–15.8)	0.863
Hemoglobin level at the last visit (mg/dL)	15.2 (13.1–15.6)	15.4 (13.7–16.2)	0.550
Baseline platelet count (**×**10^9^/L)	219 (181–260)	215 (174–258)	0.899
Platelet count at the last visit (**×**10^9^/L)	212 (176–268)	222 (184–258)	0.928
Number of implants	8 (5–8)	2 (1–4)	0.899
1	2 (13%)	13 (30%)	0.286
2	3 (20%)	5 (12%)	
3	2 (13%)	4 (9%)	
4	5 (33%)	7 (16%)	
5	0	3 (7%)	
6	1 (7%)	1 (2%)	
7	1 (7%)	0	
8	1 (7%)	9 (21%)	
10	0	1 (2%)	
Implant diameter (mm)	3.8 (3.8–4.5)	3.8 (3.8–3.8)	0.050
Implant age (months)	45 (42–56)	29 (20–46)	0.001
Implant prosthesis “all‐on‐4”	0	14 (33%)	0.012
Probing pocket depth (mm)	5.0 (3.0–7.0)	3.0 (1.0–4.0)	0.0001
Mucositis	10 (67%)	26 (61%)	0.764

*Note*: Data are presented as median and interquartile ranges or frequencies and percentages. HIV: human immunodeficiency virus.

However, the CD4+/CD8+ ratio at the time of implant placement was correlated with the presence of mucositis. Table 3 shows the comparisons between patients with and without mucositis in individual variables. The CD4+/CD8+ ratio significantly correlated with mucositis (*p* = 0.037). This means that patients' immunological profiles correlated with mucositis, and as the CD4+/CD8+ ratio decreases (specifically when the value falls below 1), the probability of mucositis increases.

The variable “number of implants” significantly correlated with mucositis (*p* = 0.0002), implying that the probability of mucositis increases as the number of implants increases.

The variable ‘implant age’ also significantly correlated with mucositis (*p* = 0.003), implying that as the implant age increases, the probability of mucositis also increases, as shown in Table 4.

**TABLE 4:** Patient characteristics based on the presence of mucositis

Characteristic	Mucositis	Healthy	*p*‐value
	(*n* = 36)	(*n* = 22)	
Age (years)	55 (51–60)	55 (49–57)	0.324
Male sex	29 (81%)	15 (68%)	0.350
Years of HIV infection	19.1 (9.5–22.8)	19.3 (10.3–29.8)	0.486
Years of antiretroviral therapy	17.3 (8.4–20.7)	18.8 (10.2–24.4)	0.218
Baseline HIVRNA >50 cp/mL	0	1 (5%)	0.379
HIVRNA >50 cp/mL at the last visit	1 (3%)	0	0.999
Baseline CD4+ level (cells/μL)	659 (588–1030)	687 (593–895)	0.836
CD4+ level at the last visit (cells/μL)	716 (518–1016)	727 (509–1000)	0.849
Mean CD4+ level during follow‐up (cells/μL)	766 (574–1022)	729 (509–957)	0.519
Baseline CD8+ level (cells/μL)	922 (641–1468)	706 (552–1073)	0.212
CD8+ at the last visit (cells/μL)	918 (545–1264)	810 (533–1080)	0.488
Baseline CD4+/CD8+ ratio	0.72 (0.67–0.90)	1.02 (0.77–1.39)	0.037
CD4+/CD8+ ratio at the last visit	0.86 (0.63–1.12)	0.87 (0.67–1.24)	0.672
Baseline hemoglobin level (mg/dL)	14.8 (13.6–15.6)	15.3 (14.8–15.8)	0.065
Hemoglobin level at the last visit (mg/dL)	15.3 (12.9–16.1)	15.4 (15.0–15.7)	0.240
Baseline platelet count (**×**10^9^/L)	212 (168–264)	229 (193–258)	0.464
Platelet count at the last visit (**×**10^9^/L)	219 (178–263)	229 (199–261)	0.947
Number of implants	8 (5–8)	2 (1–4)	0.0002
1	4 (11%)	11 (50%)	0.056
2	4 (11%)	4 (18%)	
3	4 (11%)	2 (9%)	
4	9 (25%)	3 (14%)	
5	2 (6%)	1 (5%)	
6	2 (6%)	0	
7	1 (3%)	0	
8	9 (25%)	1 (5%)	
10	1 (3%)	0	
Implant diameter (mm)	3.8 (3.8–4.5)	3.8 (3.8–3.8)	0.595
Implant age (months)	43 (27.5–50)	27 (17–38)	0.003
Implant prosthesis “all‐on‐4”	10 (28%)	4 (18%)	0.533
Probing pocket depth (mm)	3.0 (2.5–5.0)	2.5 (1.0–5.0)	0.521
Peri‐implantitis	10 (28%)	5 (23%)	0.764

*Note*: Data are presented as median and interquartile ranges or frequencies and percentages. HIV: human immunodeficiency virus.

### 3.1 Multivariate analysis

Multivariate logistic analysis showed that greater implant age correlated with the diagnosis of peri‐implantitis [1087 (1028–1178); *p* = 0.015]. The multivariate model was adjusted for age, sex, years of HIV infection, years of antiretroviral therapy, number of implants, implant diameter, CD4+ levels at baseline (BL), CD8+ levels at BL, CD4+/CD8+ ratio at BL, platelet count at BL, hemoglobin level at BL, and diagnosis of mucositis.

The multivariate logistic analysis also showed that greater implant age correlated with the diagnosis of mucositis [1059 (1011–1109); *p* = 0.016]. The model was adjusted for age, sex, years of HIV infection, years of antiretroviral therapy, number of implants, implant diameter, CD4+ levels at BL, CD8+ levels at BL, CD4+/CD8+ ratio at BL, platelet count at BL, hemoglobin level at BL, type of implant, and diagnosis of peri‐implantitis.

**FIGURE 1:**
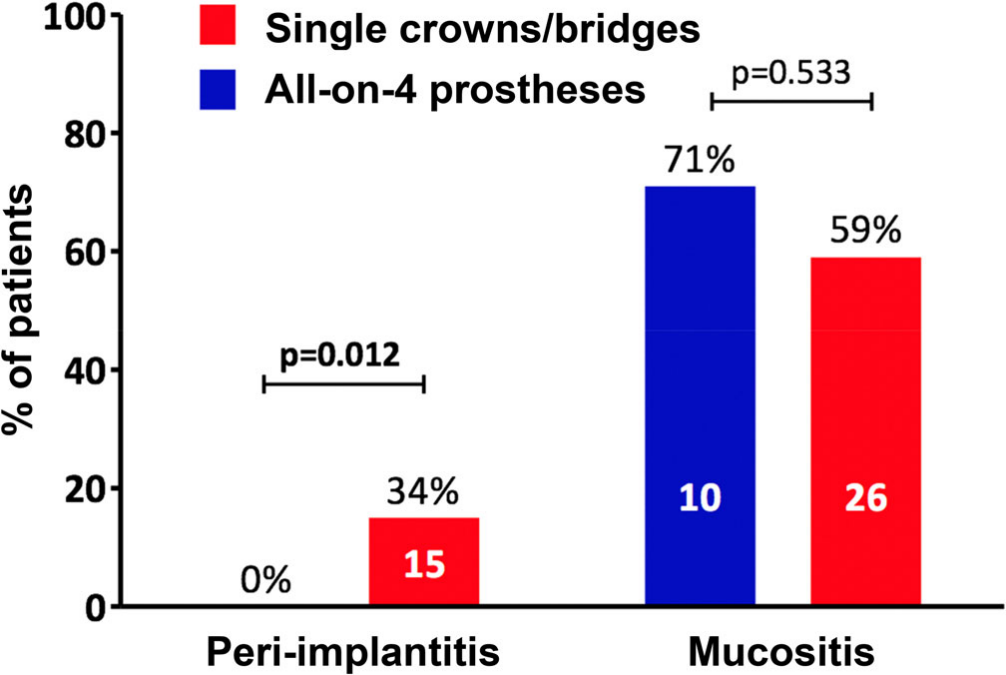
Prevalence of peri‐implantitis and mucositis in the two types of implant rehabilitation

Figure 1 shows the prevalence of peri‐implantitis in the HIV‐positive patient population. The prevalence was found to be significantly higher in patients with single crowns/bridges than that in those with all‐on‐4 prosthetic rehabilitation (*p* = 0.012). The prevalence of mucositis, in contrast, was higher in patients with all‐on‐4 prosthetic rehabilitation than in those with single crowns/bridges, but the differences were not statistically significant.

**FIGURE 2:**
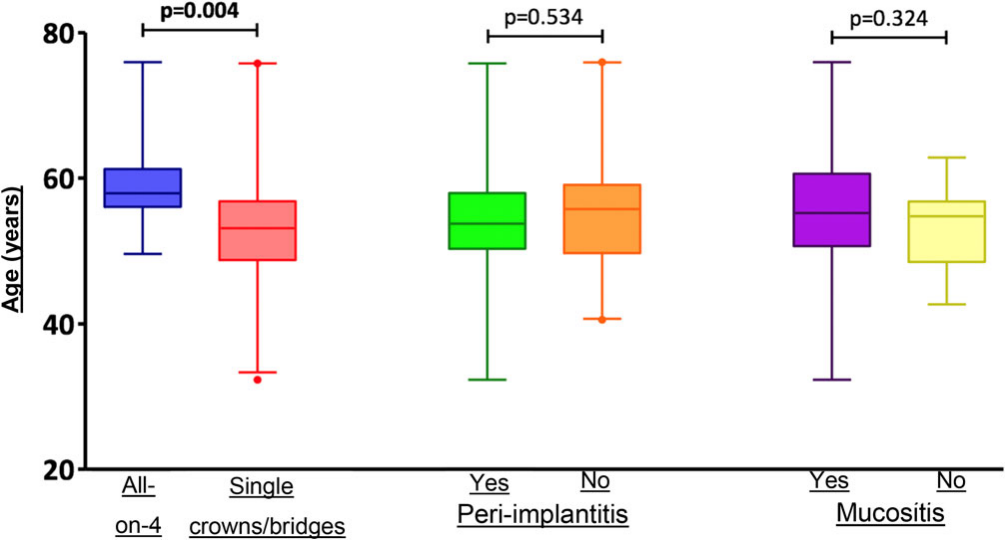
Relation between patient age and type of implant and the presence of peri‐implantitis and mucositis

Figure 2 shows the relationship of patient age with the variables “type of prosthesis” (all‐on‐4, single crowns/bridges), ‘peri‐implantitis’, and ‘mucositis’. No correlation was observed between patient age and peri‐implantitis and mucositis, while it significantly correlated with the type of prosthesis (*p* = 0.004), as patients with all‐on‐4 prosthetic rehabilitation were significantly older than those with single crowns/bridges.

**FIGURE 3:**
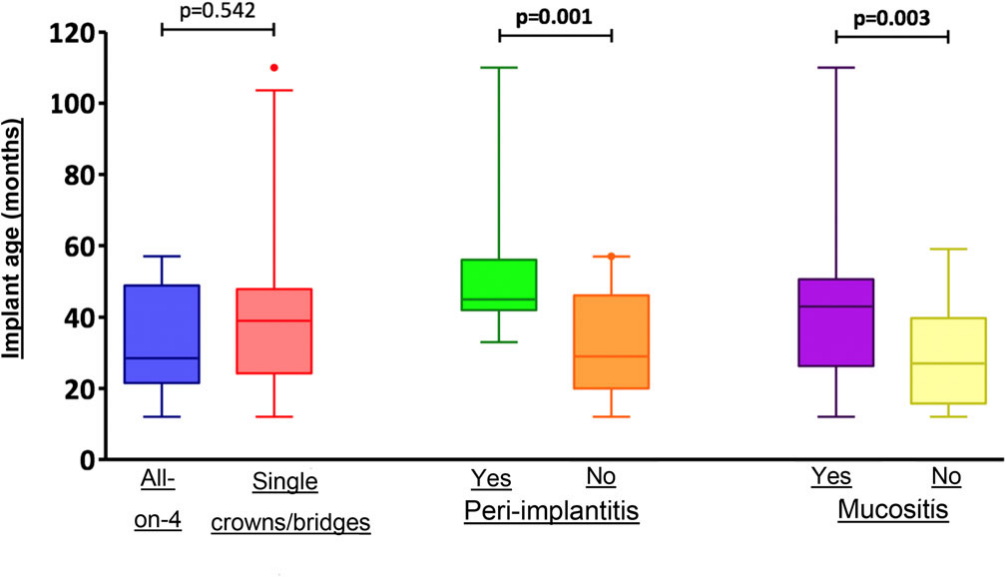
Relation between implant age and the type of implant rehabilitation and the presence of peri‐implantitis and mucositis

Figure 3 shows the relationship of implant age with the variables “type of prosthesis” (all‐on‐4, single crown/bridge), ‘peri‐implantitis’, and ‘mucositis’. No correlation was evident between implant age and type of prosthesis. In contrast, peri‐implantitis and mucositis significantly correlated with implant age, as with increasing implant age, the probability of peri‐implantitis (*p* = 0.001) and mucositis (*p* = 0.003) also increases.

**FIGURE 4:**
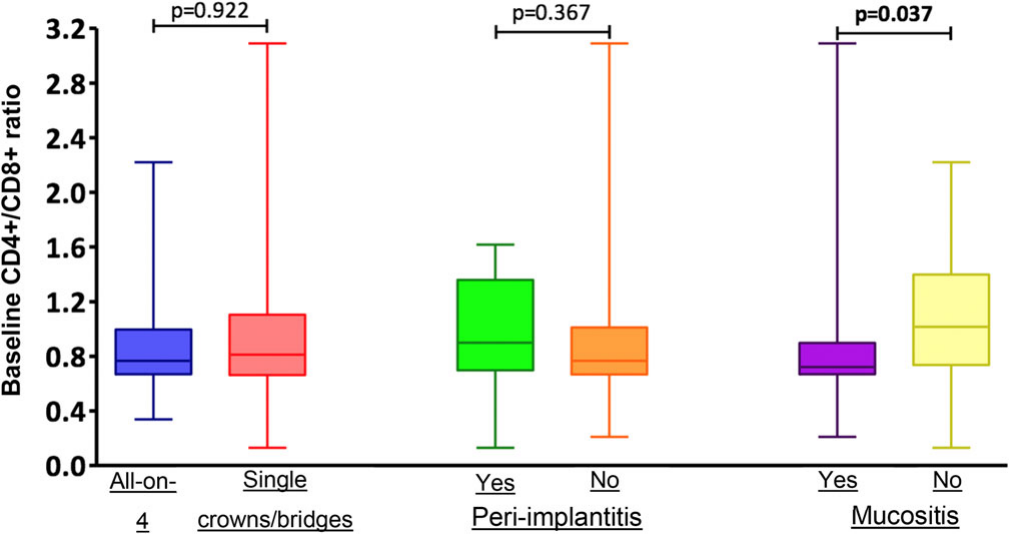
Relation between baseline CD4+/ CD8+ ratio and implant type and the presence of peri‐implantitis and mucositis

Figure 4 shows the relationship of CD4+/CD8+ ratio with the variables “type of prosthesis” (all‐on‐4, single crown/bridge), “peri‐implantitis,” and “mucositis.” No correlations were observed between type of prosthesis and CD4+/CD8+ ratio, and between peri‐implantitis and CD4+/CD8+ ratio. However, mucositis significantly correlated with CD4+/CD8+ ratio (*p* = 0.037); as this ratio decreases, the probability of mucositis increases.

### 3.2 Prevalence of peri‐implantitis and mucositis according to type of implant

Of the 222 implants, 18 (8%) exhibited peri‐implantitis and 68 (31%) exhibited mucositis. Additionally, 122 of the 222 implants (55%) were restored using single crowns/bridges, and 100 (45%) using all‐on‐4 prostheses. Of the 122 implants for single crowns/bridges, 18 (15%) exhibited peri‐implantitis, whereas 42 (34%) exhibited mucositis. None of the 100 implants for all‐on‐4 prostheses exhibited peri‐implantitis, but of the 100 implants for all‐on‐4 prostheses, 26 (26%) exhibited mucositis.

## 4 DISCUSSION

The prevalence of peri‐implantitis in HIV‐positive patients treated at the department of dentistry of our hospital (58 patients with 222 implants) was 26% and that of mucositis was 36%. Only one study has specifically analyzed the prevalence of peri‐implantitis in HIV‐positive patients (Capparé et al., 2019). However, it only compared the prevalence of peri‐implantitis between all‐on‐4 implant‐prosthetic restorations for HIV‐positive patients and controls over time for 7 years (Capparè et al., 2019). Peri‐implantitis was observed in 2 of 24 patients with a prevalence of 8.7% and in 5 of 116 implants with a prevalence of 4.3%. However, these results should be interpreted with caution, because no specific criteria were established for the diagnosis of peri‐implantitis (clinical and radiographic criteria) (Capparé et al., 2019). In our study, the prevalence of peri‐implantitis in patients with all‐on‐4 prosthetic rehabilitation was 0%. However, considering the shorter follow‐up period of our study compared with that of the study by Capparè et al. (28.5 months vs. 84 months) and higher prevalence of mucositis in implants for all‐on‐4 prostheses compared with that in implants for single crowns/bridges (71% vs. 26%), the progression of mucositis to peri‐implantitis in patients with all‐on‐4 prosthetic rehabilitation in the future is possible (Capparé et al., 2019).

Indeed, Schwarz et al. (2018) have clarified that mucositis can progress to peri‐implantitis by a mechanism similar to the progression of gingivitis to periodontitis. This process, which is influenced by oral hygiene maintenance and plaque accumulation, has not been completely understood (Costa et al., 2012). This should be evaluated through future studies conducted in the same population of HIV‐positive patients included in this study.

The prevalence of peri‐implantitis in individuals not affected by HIV has been evaluated previously (Derks & Tomasi, 2015). However, the results here should also be evaluated with caution due to the different methodologies used for the diagnosis of peri‐implantitis (Vignoletti et al., 2019). The systematic review by Derks and Tomasi in 2015 showed that the prevalence of peri‐implantitis reported in various studies ranges between 1% and 47% (Derks & Tomasi, 2015). The findings of our study fall within this reference range (26%).

Vignoletti et al. (2019) reported the prevalence of peri‐implantitis and mucositis as 35% and 38%, respectively, in a population of non‐HIV‐positive patients (237 patients with 831 implants). The presence of bleeding on probing and/or suppuration with a radiographic bone loss <2 mm and ≥ 2 mm were the diagnostic criteria for mucositis and peri‐implantitis, respectively. In contrast, the threshold for bone loss in the diagnosis of peri‐implantitis and mucositis was 3 mm in our study. Furthermore, the number of enrolled patients (831 vs. 58) and follow‐up period (38 months vs. 56 months) were lower in our study. These differences in study parameters could be the reason for the differences in the prevalence of peri‐implantitis (26% vs. 35%); the reported prevalence of mucositis was similar between the two studies (36% vs. 38%).

The secondary objective of this study was the evaluation of correlation between the immunological profile and peri‐implant pathology. No correlations were found between the prevalence of peri‐implantitis and immunological and serological profiles of patients. However, a correlation was found between the CD4+/CD8+ ratio and mucositis. Univariate analysis (Table 3) showed that CD4+/CD8+ ratio was significantly lower in patients with mucositis than that in patients without mucositis (*p* = 0.037), and as this value decreased, in particular as this value became less than 1, the probability of mucositis increased. This ratio is a diagnostic immunological marker used in numerous studies to evaluate the disease progression in HIV‐positive patients (Antinori et al., 2018). CD4+ (T‐helper) and CD8+ (T‐suppressor) are two phenotypes of T‐lymphocytes, characterized by distinct markers on surface and of different functions.

A CD4+/CD8+ ratio between 1.5 and 2.5 is usually considered normal, but heterogeneity according to sex, genetic factors, and infections is observed (McBride & Striker, 2017). In fact, a decrease in this ratio is associated with cytomegalovirus and sexually transmitted infections. HIV‐positive homosexual patients are at greater risk of sexually transmitted diseases such as syphilis, gonorrhea, viral hepatitis, and chlamydia and herpes virus infections. These diseases have been correlated with a decrease in CD4+/CD8+ ratio and an increase in CD8+ lymphocytes (Freeman et al., 2016; Pope et al., 1994; Strindhall et al., 2013; Vieira et al., 2017). The results of various studies in this regard have been conflicting, with some associating a decrease in this ratio in HIV‐positive patients with greater morbidity and mortality (Ferguson et al., 1995; Verboeket et al., 2020; Wikby et al., 1998)) and others reporting no correlation between the two (McBride & Striker, 2017).

The inversion of the CD4+/CD8+ ratio occurs in response to HIV infection in patients not treated with antiretroviral therapy and can be explained as an attempt by the host immune system to compensate for the loss of CD4+ lymphocytes through the increased production of CD8+ lymphocytes.

Initiation of antiretroviral therapy lowers the viral count but does not, in all cases, restores the CD4+/CD8+ ratio (McBride & Striker, 2017).

Capparè (2019) correlated the decrease in this ratio with long‐term implant failure including peri‐implantitis, which was defined as progressive bone loss in the presence of infection. This datum is significant, because in our study, the presence of mucositis was found to be associated with a decrease in CD4+/CD8+ ratio in a much lower follow‐up period (38 months vs. 84 months) (Capparé et al., 2019). Mucositis, under certain conditions, might progress to peri‐implantitis, if further follow‐ups and investigations are conducted (Schwarz et al., 2018). If this occurs, the correlation between decrease in CD4+/CD8+ ratio and peri‐implantitis reported in the aforementioned study would be confirmed (Capparé et al., 2019). In the peri‐implant mucosal system, the host response includes both vascular and cellular elements, and T‐lymphocytes are usually present in small groups in the connective tissue lateral to the epithelial attachment (Crespi et al., 2012). Mucositis is characterized by inflammatory infiltrate rich in vascular elements, plasma cells, and lymphocytes, which does not extend into the connective tissue above the bone crest and is confined to the level of the junctional epithelium (Berglundh et al., 2018). Therefore, a disorder in the lymphocytic inflammatory response may influence the prevalence of peri‐implant disease.

Another secondary objective was to evaluate the differences among types of implant‐prosthetic rehabilitation with respect to peri‐implant disease and the variables analyzed.

The patients were divided into 2 groups according to the type of prosthetic rehabilitation: patients with all‐on‐4 prostheses and those with single crowns/bridges.

The all‐on‐4 method of implant‐prosthetic treatment was introduced by Malò in 2003 and is a widely approved treatment modality with good clinical results (Agliardi, Panigatti, et al., 2010; Agliardi, Clerico, et al., 2010; Crespi et al., 2012). This type of implant‐prosthetic rehabilitation involves the placement of 4 implants in the anterior maxilla or in the inter‐foraminal region of the mandible ( Gherlone et al., 2016a; Gherlone et al., 2016b). Of these 4 implants, the 2 distal‐most implants are inclined distally and the 2 mesial implants are straight to support a full‐arch prosthesis with a molar cantilever of variable size ( Gherlone et al., 2016a; Gherlone et al., 2016b; Penarrocha‐Diago et al., 2017).

In our study, of the 222 implants, 122 were rehabilitated using single crowns/bridges (43 patients) while 100 were rehabilitated using all‐on‐4 prostheses (15 patients).

Of the two groups, patients with single crowns/bridges showed a prevalence of peri‐implantitis higher (34%) than patients restored using all‐on‐4 prostheses (0%), and the difference was statistically significant (*p* = 0.012). This finding should be interpreted carefully, due to the differences in the number of patients and implant age between the two groups (12 months), that is patients with all‐on‐4 prostheses had implants 1‐year‐younger than patients with single crowns/bridges. However, the data can also be partly explained by the fact that all‐on‐4 prosthetic rehabilitations are performed using a specific clinical protocol that includes a series of standardized steps in both surgical and prosthetic phases. In addition, the all‐on‐4 rehabilitations were performed by an expert in this method of implant‐prosthetic rehabilitation, and were periodically disassembled and assessed, with institution of professional hygiene measures.

However, peri‐implantitis has been described as a complication of implant treatment even with all‐on‐4 prosthetic treatments. Peñarrocha‐Diago et al. have described peri‐implantitis as the second most common biological complication of all‐on‐4 prosthetic rehabilitations (Penarrocha‐Diago et al., 2017).

Malò et al. evaluated all‐on‐4 implant‐prosthetic rehabilitations on 1884 implants in 471 patients over a follow‐up period between 10 and 18 years (Maló et al., 2019). They evaluated the prevalence of peri‐implant disease (mucositis and peri‐implantitis), which was defined as the presence of pockets ≥5 mm around the peri‐implant tissue with bleeding on probing and bone loss assessed by probing using a millimeter periodontal probe. They found that peri‐implant disease (unspecified) was present in 108 patients (23%) and 207 implants (11%).

The comparison among various studies in the literature is often difficult, because the type of prosthesis used to restore the implant and the criteria for the diagnosis of peri‐implantitis are not clearly defined.

The findings of this study showed that patients rehabilitated using all‐on‐4 implant‐prosthetic restorations were significantly older than those rehabilitated using single crowns/bridges (*p* = 0.004). This could be attributed to the facts that all‐on‐4 rehabilitations are performed in cases of bone atrophy and that the degree of bone atrophy is correlated with loss of teeth and precisely to patient's age (Agliardi, Clerico, et al., 2010; Agliardi, Panigatti, et al., 2010; Penarrocha‐Diago et al., 2017). The number of implants used in a patient often correlates with peri‐implantitis (Derks et al., 2016; Vignoletti et al., 2019). In this study as well, this parameter appears to have the greatest correlation with peri‐implantitis and mucositis, according to the multivariate analysis.

This study has several limitations. The presence of plaque and history of periodontitis are strongly associated with peri‐implantitis; however, it was not possible to evaluate the presence of plaque and periodontal status of the same patient. Therefore, periodontally healthy patients could not be differentiated from periodontitis patients and patients with good oral hygiene could not be distinguished from those with poor oral hygiene.

From the available data and within the limitations of this study, we found that the prevalence of peri‐implantitis was 26% in the population of HIV‐positive patients and 8% in the dental implants. No correlation was found between patients' immunological and serological factors and peri‐implantitis. However, mucositis was found to be associated with CD4+/CD8+ ratio, which was found to be an immunological risk factor for mucositis. The most important risk factor for peri‐implantitis and mucositis was implant age.

## CONFLICT OF INTEREST

The authors declare no conflict of interest.

## AUTHOR CONTRIBUTION

Luca Casula contributed to conception, design, data acquisition and interpretation, drafted and critically revised the manuscript. Andrea Poli performed all the statistical analysis. Giulia Artuso contributed to design, data acquisition and interpretation. Paolo Capparé contributed to conception, drafted and critically revised the manuscript. Enrico F. Gherlone contributed to conception, drafted and critically revised the manuscript. All authors gave their final approval and agree to be accountable for all aspects of the work.

## ETHICS APPROVAL STATEMENT

All procedures were approved by the Ethics Committee of University Vita‐Salute, San Raffaele Hospital, Milan (on 15/7/2020 with number of protocol “PERIHIV2”—EC Reg. N. 133/INT/2020).

## PATIENT CONSENT STATEMENT

Appropriate informed consent forms were provided to all patients and were signed if the patients agreed to participate in the study.

## PERMISSION TO REPRODUCE MATERIAL FROM OTHER SOURCES

NOT Applicable because all the material is product by the author of this article in University Vita‐Salute, San Raffaele Hospital.

## CLINICAL TRIAL REGISTRATION

ClinicalTrials.gov Identifier: NCT04829968.

## Supporting information


**Appendix S1:** Supplementary Information

## Data Availability

Data statement file is available and uploaded as PDF file called “Data statement”
