# Supplementary material for: Prevalence of peri‐implantitis in a sample of HIV‐positive patients
Source: Clin Exp Dent Res. 2021 Jul 20;7(6):1002–13. doi: 10.1002/cre2.469 (PMC8638315; doi:10.1002/cre2.469)
Supplement: Supplementary file 1 — Appendix S1: Supplementary Information [file CRE2-7-1002-s001.zip › CRE2_469_CRE2_469_cre2.20210173-File002.pdf]

ID	Gender	N_IMP	Pos_imp	PPD	BoP_Pus	Diametro_impiant	MAX_DIAN	Eta_Impiant	Num_impiant	Tipo_ProteS	N_PER	Peri_implantit	N_MUCO	MucoSite	CD4_BL	LAST_CD4	CD4m	CD8_BL	LAST_CD8	HIVRNA_BI	LAST_HIVRN	HB_BL	LAST_HB	PLT_BL	LAST_PLT	age	years_hiv	years_art	CD4_CD8_BL	LAST_CD4_CD	HIVPOS_BI	LAST_HIVPOS
1	F	1	16	7	Si	3,8	3,8	47	1	Singola	1	Si	0	No	1244	1000	1248	769	533	0,9	0,9	12,3	13,2	193	167	53,9657769	28,7556468	24,3531828	1,61768531	1,876172608	0	0
2	F	4	15	2	No	3,8	3,8	27	8	All on 4	0	No	0	No	682	717	729	490	461	0,9	0,9	14,9	15,4	207	236	56,1752225	28,0848734	28,0848734	1,39183673	1,555314534	0	0
3	M	8	15	2	No	3,8	3,8	54	8	All on 4	0	No	1	No	705	732	705	961	1173	0,9	39	13,4	13,5	179	206	60,9089665	27,835729	21,2977413	0,73361082	0,624040921	0	0
4	F	8	15	1	No	3,8	3,8	22	8	All on 4	0	No	1	No	1399	1043	1004	1502	998	0,9	0,9	14,3	14,7	270	258	57,4948665	27,3347023	22,9212868	0,93142477	1,04509018	0	0
5	F	1	46	1	No	3,3	3,3	29	1	Singola	0	No	0	No	420	456	450	642	477	0,9	0,9	16,9	15,9	95	101	55,7289528	34,0835044	28,6707734	0,65420561	0,955974843	0	0
6	F	3	46	1	No	3,8	3,8	27	3	Ponte	0	No	0	No	637	745	779	833	600	39	39	14,2	13,7	201	202	56,8213552	29,8370979	27,5427789	0,76470588	1,241666667	0	0
7	M	8	15	3	No	3,3	3,8	57	8	All on 4	0	No	1	No	148	63	114	438	136	0,9	39	13	9,3	36	355	59,3456537	36,0848734	17,8562628	0,33789954	0,463235294	0	0
8	F	1	46	3	Si	3,8	3,8	46	1	Singola	0	No	1	Si	591	700	700	828	644	0,9	0,9	12,8	12,7	215	255	59,8631075	34,0835044	22,1629021	0,71376812	1,086956522	0	0
9	M	4	45	1	No	3,8	3,8	22	4	All on 4	0	No	0	No	995	1254	1157	1073	1531	0,9	0,9	16,8	17,4	214	280	57,4291581	35,08282	22,8364134	0,92730662	0,819072502	0	0
10	M	7	15	3	Si	3,8	3,8	51	8	All on 4	0	No	1	Si	957	1632	1695	1433	1857	0,9	0,9	14,5	15,7	182	201	55,8384668	22,5270363	22,0835044	0,66782973	0,878836834	0	0
11	M	2	16	5	Si			23	2	Singola	0	No	1	Si	1024	963	903		788	0,9	0,9	16,2	17	174	184	52,6078029	20,9993155	20,1724846		1,222081218	0	0
12	F	4	35	3	No	3,8	3,8	56	4	Ponte	0	No	1	No	294	495	416	590	675	0,9	0,9	12,5	12,4	254	247	52,4681725	25,4182067	25,4182067	0,49830508	0,733333333	0	0
13	M	5	24	3	No	3,8	3,8	39	5	Ponte	0	No	1	No	588	506	607		1511	0,9	39	11,2	11	137	253	75,578371	22,587269	19,7535934		0,334877565	0	0
14	F	4	15	1	No	3,8	3,8	38	4	All on 4	0	No	0	No	1226	1247	1486	552	516	0,9	0,9	15,3	15,1	144	165	58,3271732	35,08282	31,5482546	2,22101449	2,416666667	0	0
15	M	8	46	7	No	3,8	3,8	110	8	Ponte	1	Si	1	No	410	415	513	1213	1138	0,9	0,9	14,7	12,5	254	427	75,7864476	21,2511978	21,1690623	0,33800495	0,364674868	0	0
16	M	1	24	3	No	3,8	3,8	17	1	Singola	0	No	0	No		430	456		1001		0,9	15	15		153	48,9144422	30,0835044	28,0848734		0,42957043	0	0
17	M	1	15	5	Si pus	4,5	4,5	45	4	Singola	0	No	1	Si	1431	1211	1346	2116	1643	0,9	39	15,7	15,9	290	325	51,6687201	21,7549624	21,5687885	0,67627599	0,737066342	0	0
18	M	8	15	1	No	3,8	3,8	30	8	All on 4	0	No	1	No	1030	826	898	1537	1264	39	0,9	15,3	16,6	313	236	59,430527	22,9295003	22,6036961	0,67013663	0,653481013	0	0
19	M	4	45	3	Si	4,5	4,5	25	4	Ponte	0	No	1	Si	629	668	670	622	637	0,9	39	14,6	15,8	157	148	54,4914442	35,08282	18,9623546	1,01125402	1,04866562	0	0
20	M	7	14	1	No	3,3	3,8	33	7	Singola	1	No	1	No	657	887	758	941	1426	0,9	204	15,5	15,2	217	239	52,7446954	20,3367556	19,0390144	0,69819341	0,622019635	0	1
21	M	8	15	2	Si	3,8	3,8	48	8	All on 4	0	No	1	Si	388	608	595	654	875	0,9	0,9	16,1	16,3	174	159	69,2375086	27,08282	23,5017112	0,59327217	0,694857143	0	0
22	M	4	35	5	Si	3,8	3,8	38	4	Ponte	1	No	1	Si	575	716	721	729	972	0,9	0,9	16,7	17,1	145	144	71,6605065	20,4188912	20,2272416	0,78875171	0,736625514	0	0
23	F	1	46	1	No	3,8	3,8	12	1	Singola	0	No	0	No	622	509	509	542	691	0,9	0,9	13	13	230	229	55,0855578	19,5865845	19,4086242	1,14760148	0,736613603	0	0
24	M	2	14	5	No	3,8	3,8	45	2	Ponte	1	Si	0	No		727	677		1080		39	15,1	15,1		212	51,9014374	22,5023956	19,6303901		0,673148148	0	0
25	M	1	46	5	No	3,8	3,8	12	1	Singola	0	No	0	No	869	1048	1044	612	624	0,9	0,9	15,8	15,6	250	251	56,7665982	18,5571526	17,8507871	1,41993464	1,679487179	0	0
26	F	2	46	2	No	3,8	3,8	18	2	Ponte	0	No	0	No		1045	957		963	0,9	39	15,3	15,5	176	194	53,431896	36,0848734	23,08282		1,085150571	0	0
27	M	3	15	1	No			48	3	Singola	0	No	1	No	864	470	539		742	0,9	0,9	15,3	15,1	233	204	48,6488706	17,9411362	8,75290897		0,633423181	0	0
28	M	3	13	2	No	3,8	3,8	19	3	Singola	0	No	0	No	840	645	769	1593	1407	39	0,9	15,3	15,4	276	272	51,5537303	19,0554415	18,78987	0,52730697	0,458422175	0	0
29	M	4	24	5	Si	3,8	4,5	56	4	Ponte	1	No	1	Si	596	648	582		386	0,9	39	15,2	15,4	265	302	53,7303217	17,1416838	16,7748118		1,678756477	0	0
30	M	8	25	1	Si	3,8	3,8	24	8	All on 4	0	No	1	Si	1613	1466	1590	1520	1677	0,9	39	14,1	13,7	257	269	62,2587269	31,08282	18,6310746	1,06118421	0,874180083	0	0
31	M	1	26	2	No	3,8	3,8	29	1	Singola	0	No	0	No	622	491	548	564	462	0,9	0,9	17,1	16,6	187	201	62,8227242	15,8521561	14,8829569	1,10283688	1,062770563	0	0
32	F	4	45	5	Si	3,8	3,8	56	4	Ponte	1	No	1	Si	655	791	773	482	468	0,9	0,9	11,7	13,1	407	284	51,7700205	19,08282	18,5023956	1,35892116	1,69017094	0	0
33	M	5	46	1	No	3,8	3,8	12	5	All on 5	0	No	0	No	395	429	429	515	711	39	39	14,5	15,3	258	292	54,3819302	21,0841889	14,8391513	0,76699029	0,603375527	0	0
34	M	2	11	5	Si	3,8	3,8	30	2	Ponte	0	No	1	Si	493	461	523	731	545	0,9	0,9	15,8	16,3	266	318	51,8329911	13,9164956	10,8336756	0,6744186	0,84587156	0	0
35	M	4	45	1	No	3,8	3,8	20	4	All on 4	0	No	1	No		1030	1287		918	39	42	14,4	13,8	163	147	56,1149897	12,8898015	12,4626968		1,122004357	0	0
36	M	8	15	3	Si	3,8	3,8	48	8	All on 4	0	No	1	Si	653	444	468	845	401	39	0,9	14,8	11,9	244	176	75,9507187	12,9965777	12,5585216	0,77278107	1,10723192	0	0
37	M	1	36	5	No	3,8	3,8	37	1	Singola	0	No	0	No	692	760	840	825	872	0,9	0,9	14,6	15,6	250	280	46,6885695	13,982204	11,2744695	0,83878788	0,871559633	0	0
38	F	6	36	3	No	4,5	4,5	24	6	Singola	0	No	1	No	403	449	437	627	473	0,9	39	12,7	12	208	217	49,6783025	11,797399	11,6030116	0,64274322	0,949260042	0	0
39	M	2	24	7	Si	3,8	4,5	59	4	Ponte	1	Si	0	No	895	794	758	613	811	0,9	39	15,6	14,8	250	235	55,1512663	10,2505133	10,1984942	1,46003263	0,979038224	0	0
40	F	2	14	7	Si	3,8	3,8	39	2	Ponte	1	Si	1	No		551	879		335		39	12,3	12,3		203	57,9356605	9,72210815	9,26214921		1,644776119	0	0
41	M	5	44	3	Si	3,8	3,8	53	5	Ponte	0	No	1	Si	1128	806	841	365	247	0,9	39	16,2	16,6	177	179	73,1088296	8,08487337	8,08487337	3,09041096	3,263157895	0	0
42	M	1	16	5	Si	3,8	3,8	19	1	Singola	0	No	1	Si	1195	1001	1036	1778	2128	0,9	0,9	13,7	13,6	160	197	57,7823409	9,21013005	6,99794661	0,67210349	0,470394737	0	0
43	M	1	46	2	No	3,8	3,8	12	1	Singola	0	No	0	No												47,1403149	9,16358658				0	0
44	M	10	15	3	No	3,8	3,8	12	10	All on 4	0	No	1	No	907	941	941	1299	1096	0,9	0,9	14,8	15,4	365	248	49,5687885	16,1697467	11,6303901	0,69822941	0,858576642	0	0
45	M	2	34	5	No	3,3	3,3	48	2	Singola	0	No	1	Si	1287	1574	1527	1584	1005	0,9	39	15,8	16,2	168	165	55,9780972	8	4,6844627	0,8125	1,566169154	0	0
46	M	1	36	3	Si	4,5	4,5	45	1	Singola	1	Si	0	No	593	657	699	463	376	0,9	0,9	16,1	15,6	220	200	56,3778234	7,99726215	7,9890486	1,28077754	1,747340426	0	0
47	M	4	45	2	No	3,8	3,8	43	6	Ponte	1	No	1	No	659	554	600	903	826	0,9	0,9	14,5	15,6	173	176	60,843258	20,0848734	20,0848734	0,72978959	0,670702179	0	0
48	M	1	46	5	No	4,5	4,5	32	1	Singola	0	No	0	No	881	645	707	960	810	0,9	39	17	17,7	301	318	45,3880903	15,08282	9,3963039	0,91770833	0,796296296	0	0
49	M	3	45	3	No	3,8	4,5	45	3	Ponte	0	No	1	No	269	530	495	1282	1625	0,9	39	16,2	16,3	264	220	41,9192334	6,88295688	7,84941821	0,20982839	0,326153846	0	0
50	M	4	32	5	Si	3,8	3,8	56	4	Ponte	0	No	1	Si	755	1295	1068	878	949	0,9	0,9	14,8	15,7	291	294	44,7802875	6,46680356	6,23956194	0,85990888	1,36459431	0	0
51	M	2	15	7	Si	3,8	3,8	55	2	Singola	1	Si	0	No	287	813	477	2212	1634	40	0,9	15,5	15,7	286	261	43,9342916	5,89459274	5,81245722	0,12974684	0,49755202	0	0
52	M	3	14	5	Si	3,8	4,5	43	3	Singola	1	No	1	Si	1415	668	1007	1573	844	0,9	39	18	17,4	188	187	32,3203285	5,58795346	5,42642026	0,89955499	0,791469194	0	0
53	F	4	35	3	No	4,5	4,5	42	4	Ponte	1	No	1	No		1258	1478		1139		39	13	13		268	50,275154	5,58521561	5,36892539		1,104477612	0	0
54	M	2	46	5	No	4,5	4,5	12	2	Singola	0	No	0	No	1661	1016	1202	1501	1188	53	39	14,8	17,4	345	199	42,7022587	5,42915811	5,41820671	1,1065956	0,855218855	1	0
55	M	1	36	3	Si	3,3	3,3	42	1	Singola	0	No	1	Si	1128	422	841	1260	2616	39	39	15	12,2	251	223	54,5544148	5,3963039	5,25667351	0,8952381	0,161314985	0	0
56	M	3	36	5	Si	3,8	4,5	49	3	Ponte	1	Si	1	No	657	715	565	621	466	0,9	0,9	14,8	15,4	127	157	40,3887748	19,08282	12,6488706	1,05797101	1,534334764	0	0
57	M	1	26	4	No	3,8	3,8	21	1	Singola	0	No	0	No	444	458	450	1923	1578	0,9	0,9	15,6	14,6	229	253	59,063655	4,79945243	4,58316222	0,23088924	0,290240811	0	0
58	M	1	36	4	Si	4,5	4,5	25	1	Singola	0	No	1	Si	1000	1121	1058			19	19	15	15,5	142	172	40,5831622	3,93976728	3,73990418			0	0

s
